# Novel ubiquitin-independent nucleolar c-Myc degradation pathway mediated by antizyme 2

**DOI:** 10.1038/s41598-018-21189-0

**Published:** 2018-02-14

**Authors:** Noriyuki Murai, Yasuko Murakami, Ayasa Tajima, Senya Matsufuji

**Affiliations:** Department of Molecular Biology, The Jikei University School of Medicine, 3-25-8 Nishi-shinbashi, Minato-ku, Tokyo 105-8461 Japan

## Abstract

The proto-oncogene *c-Myc* encodes a short-lived protein c-Myc that regulates various cellular processes including cell growth, differentiation and apoptosis. Degradation of c-Myc is catalyzed by the proteasome and requires phosphorylation of Thr-58 for ubiquitination by E3 ubiquitin ligase, Fbxw7/ FBW7. Here we show that a polyamine regulatory protein, antizyme 2 (AZ2), interacts with c-Myc in the nucleus and nucleolus, to accelerate proteasome-mediated c-Myc degradation without ubiquitination or Thr-58 phosphorylation. Polyamines, the inducer of AZ2, also destabilize c-Myc in an AZ2-dependent manner. Knockdown of AZ2 by small interfering RNA (siRNA) increases nucleolar c-Myc and also cellular pre-rRNA whose synthesis is promoted by c-Myc. AZ2-dependent c-Myc degradation likely operates under specific conditions such as glucose deprivation or hypoxia. These findings reveal the targeting mechanism for nucleolar ubiquitin-independent c-Myc degradation.

## Introduction

c-Myc is a transcription factor that regulates a variety of target genes related to cell growth, differentiation, and apoptosis as well as ribosomal RNA genes^1,2^. It is a short lived protein which is degraded by the ubiquitin-proteasome pathway^3,4^. Two phosphorylation sites, Thr-58 and Ser-62, are critical for ubiquitination of c-Myc, and the phosphorylated Thr-58 is directly recognized by E3 ubiquitin ligase, Fbxw7 (in mouse, FBW7)^5,6^. Proteasome inhibition causes accumulation of c-Myc in nucleoli, suggesting that a certain part of c-Myc is degraded in the nucleolus^7^.

Polyamines are bioactive substances required for cell growth and regulate various cellular processes such as differentiation, apoptosis, cell signaling and protein synthesis^8^. Three major polyamines, putrescine, spermidine and spermine, are present in mammalian cells. Intracellular polyamines are tightly regulated by proteins termed antizyme (AZ). AZ is induced by polyamines through ribosomal frameshifting^9^. AZ represses ornithine decarboxylase (ODC), a key enzyme for polyamine synthesis, and inhibits cellular uptake of polyamines, and thus provides the feedback regulation for the cellular polyamine level (reviewed in ref.^10^). Mammalian cells express three members of the AZ family (AZ1-3)^11,12^ (reviewed in ref.^13^). AZ1 and AZ2 are distributed in most tissues whereas AZ3 expressed only in testis^13^. AZ1 and AZ2 are different. AZ2 binds to ODC but less tightly than does AZ1. Both AZ1 and AZ2 accelerate ODC degradation in living cells^10,14,15^ but only AZ1 exerts this activity in cell-free systems such as reticulocyte lysates and cell extracts^16^. We found that AZ1 mainly distributes in the cytoplasm whereas a proportion of AZ2 localizes in the nucleus^17,18^. Clinically it has been reported that a high expression of AZ2 mRNA, but not of AZ1 mRNA, correlates with increased survival of neuroblastoma patients^19^.

To find a clue to an AZ2-specific role, we explored novel AZ2-interacting molecules by yeast two-hybrid screening. Using mouse AZ2 cDNA as a bait we identified a leucine zipper protein that interacts with c-Myc as a candidate for an AZ2-interacting protein from a mouse brain cDNA library (Murai *et al*., manuscript in preparation). During subsequent analysis for a ternary interaction involving the candidate proteins, AZ2, and c-Myc, we serendipitously found that AZ2 directly binds to c-Myc. Here we show evidence that AZ2 accelerates c-Myc degradation in an ubiquitin-independent manner and affects the pre-rRNA synthesis by regulating c-Myc level in the nucleolus. This novel c-Myc degradation pathway is induced under glucose deprivation or hypoxia conditions.

## Results

### AZ2 interacts with c-Myc in the nucleus and nucleolus

**Figure 1:**
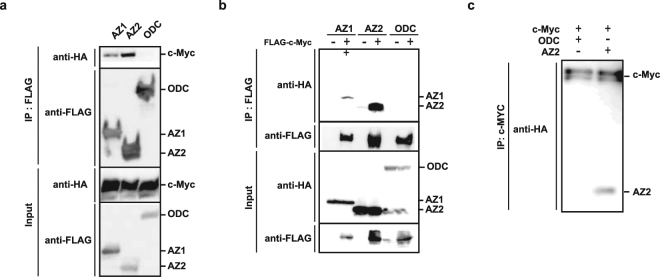
Interaction between AZ2 and c-Myc. (**a**) cDNA for HA-c-Myc was transfected with either FLAG-AZ1, FLAG-AZ2 or FLAG-ODC in 293-F cells. Cell lysates were immunoprecipitated with anti-FLAG antibody (IP: FLAG) and the resulting precipitates as well as the original cell lysates (input), were subjected to immunoblot analysis with anti-HA or anti-FLAG antibody. The experiment was repeated three times (**b**) The above experiment was performed with swapped tags (HA-AZ1, HA-AZ2, HA-ODC and FLAG-c-Myc). Immunoprecipitaion was performed with anti-FLAG antibody and c-Myc bound proteins were detected with anti-HA antibody. Expressed protein levels in cell lysates were checked by immunoblotting with anti-HA antibody. Experiments were repeated three times. (**c**) *In vitro* immunoprecipitation assay was performed using Human HA-AZ2, HA-c-Myc or HA-ODC purified from 293-F cells. Purified proteins were mixed in M-PER buffer, and after overnight incubation at 4 °C, HA-c-Myc was immunoprecipitated by anti-c-Myc antibody and c-Myc bound protein was detected by immunoblotting using anti-c-Myc antibody. Detailed protocol for *in vitro* immunoprecipitation assay indicated in Methods.

**Figure 2:**
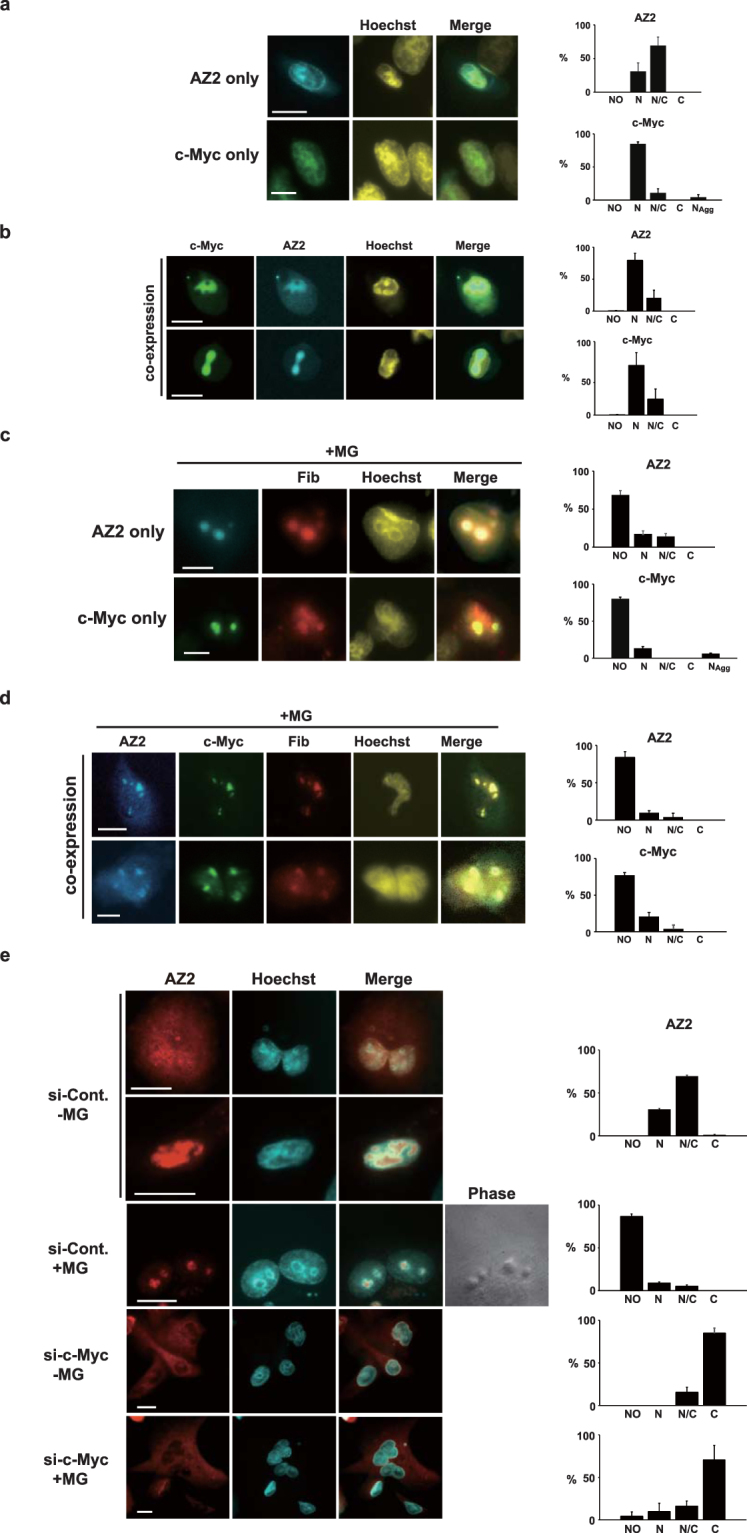
Subcellular localization of c-Myc and AZ2 (**a**) Panc-1 cells were transiently transfected with either ECFP-AZ2 or EYFP-c-Myc or both, cultured for 24 h, and observed under a fluorescence microscopy. Immunofluorescence images show the localization of individually expressed ECFP-AZ2 (upper, AZ2 only), EYFP-c-Myc (upper, c-Myc only). Monochrome images of AZ2 and c-Myc were colored in cyan and green, respectively, and images of nuclei stained with Hoechst 33342 were colored in yellow. Nuclear dominant images of AZ2 and c-Myc are indicated. NO, nucleolar dominant distribution, N, nuclear dominant distribution; N/C, nuclear and cytosolic distribution, C, cytosolic dominant distribution. (**b**) Panc-1 cells were transiently transfected with both ECFP-AZ2 and EYFP-c-Myc. Subcellular localization of the proteins was analyzed as in (**a**). In the coexpression cells, localization of AZ2 and c-Myc was identical. (**c**) Panc-1 cells were transiently transfected with either ECFP-AZ2 or EYFP-c-Myc. After 24 h, cells were treated with 20 µM MG132 for 5 h. Then cells were fixed and subcellular localization of the proteins was analyzed as in (**a**). Nucleolar distribution was confirmed by detecting nucleolar protein, fibrillarin using anti-fibrillarin (Fib) antibody and secondary antibody, AlexaFluor555 (orange)-conjugated anti-rabbit IgG as described in methods. N_Agg_, nuclear dominant distribution with aggregated forms. (**d**) Panc-1 cells were transiently transfected with both ECFP-AZ2 and EYFP-c-Myc, and after 24 h, cells were treated with 20 µM MG132 for 5 h. Subcellular distribution of these proteins were analyzed as in (**c**). (**e**) Panc-1 cells were transfected with c-Myc siRNA or control siRNA. After 24 h, the cells were transfected with HA-AZ2 (human) and incubated for further 24 h. Then the cells were treated with MG132 for 5 h and immunostained with anti-HA antibody and secondary antibody conjugated with AlexaFluor 555. Monochrome images of AZ2 and nuclei were colored in orange and cyan, respectively. A phase-contrast image was added to confirm the location of nucleoli (siCont + MG). Bar graphs represent quantification of the cells with subcellular localization of the proteins. C, cytosolic distribution. Bar graphs on the right of each images represent quantification of 50 cells (**a–d**) or 100 cells (**e**). Data (**a–e**) shown represent the mean ± SD calculated from three independent experiments. Scale bars, 20 μm.

Figure 1a shows the result of immunoprecipitation assay using FLAG-tagged AZ1, AZ2 or ODC and hemagglutinin epitope (HA)-tagged c-Myc expressed in 293-F cells. Both AZ1 and AZ2 were coprecipitated with c-Myc, and apparently AZ2 bound to c-Myc more strongly than did AZ1. Negative controls, FLAG-ODC and only FLAG-vector, were not coprecipitated with HA-c-Myc. An immunoprecipitation assay with the swapped tags brought about essentially the same result (Fig. 1b). We further performed an *in vitro* immunoprecipitation assay using HA-AZ2, HA-c-Myc and HA-ODC affinity purified from 293-F cells (Fig. S1) with anti-HA antibody. The binding between c-Myc and AZ2 were also confirmed *in vitro* (Fig. 1c).

c-Myc is known to localize in the nucleus and nucleoli^1^. Nuclear c-Myc binds to MYC-associated factor X (MAX) forming a heterodimer through the leucine zipper and the complex binds to E-Box sequences in the promoters of various target genes^20^. Nucleolar c-Myc plays a key role in regulating ribosome biogenesis^21^. Since AZ2 is mainly localized in the nucleus^17^, we tested whether AZ2 colocalizes with c-Myc in the nucleus and/or nucleoli. AZ2 fused with enhanced cyan fluorescent protein (ECFP) and c-Myc fused with enhanced yellow fluorescent protein (EYFP) were expressed in the human pancreas carcinoma-derived cell line, Panc-1 cells and then observed under fluorescent microscopy. When expressed alone, ECFP-AZ2 dominantly distributed in the nucleus in 40% of the cells and in both cytoplasm and nucleus in the reminder (60%), whereas EYFP-c-Myc was distributed in the nucleus in more than 80% of the cells (Fig. 2a, panels and bar graphs). When ECFP-AZ2 and EYFP-c-Myc were expressed together, they colocalized in the nucleus in more than 80% of the cells (Fig. 2b, panels and bar graph). We next tested the effect of the proteasome inhibitor MG132 that is known to cause nucleolar accumulation of c-Myc^22^. As shown in Fig. 2c, when individually expressed, not only EYFP-c-Myc but also ECFP-AZ2, were largely shifted to the nucleoli in 80% of the cells 5 h after addition of MG132. In about 10% of the cells, EYFP-c-Myc was aggregated in the nucleus (N_Agg_ in bar graph, photograph not shown). Nucleolar localization of AZ2 and c-Myc was confirmed by the nucleolar marker, Fiblillarin (Fib, orange). When ECFP-AZ2 and EYFP-c-Myc were coexpressed in the presence of MG132, the two proteins were colocalized in the nucleoli in 70% of the cells (Fig. 2d). Notably, aggregated EYFP-c-Myc was not observed when ECFP-AZ2 was coexpressed. The nucleolar accumulation of proteins by MG132 is not a general phenomenon since ODC, which is known to be degraded by proteasome, stayed in the cytosol even in the presence of MG132 (Fig. S2 and bar graph). To elucidate the influence of endogenous c-Myc on the localization of AZ2, Panc-1 cells expressing HA-AZ2 were treated with c-Myc or control siRNA with or without MG132 and then immunostained with anti-HA antibody and fluorescent secondary antibody. In the cells treated with control siRNA, 30% of HA-AZ2 was distributed in the nucleus and 70% was distributed in both nucleus and cytoplasm (Fig. 2e, si-Cont -MG and bar graph). MG132 shifted the localization of AZ2 to the nucleoli in 90% of the cells (Fig. 2e, si-Cont + MG and bar graph) consistent with Fig. 2c. Knockdown of c-Myc dramatically changed the HA-AZ2 distribution to the cytoplasm both in the presence and absence of MG132 (Fig. 2e, si-c-Myc –MG and + MG and bar graph, methods). These results indicate that the nuclear and nucleolar localization of AZ2 is dependent of c-Myc.

### AZ2 accelerates proteasomal degradation of c-Myc in a manner independent of ubiquitination

**Figure 3:**
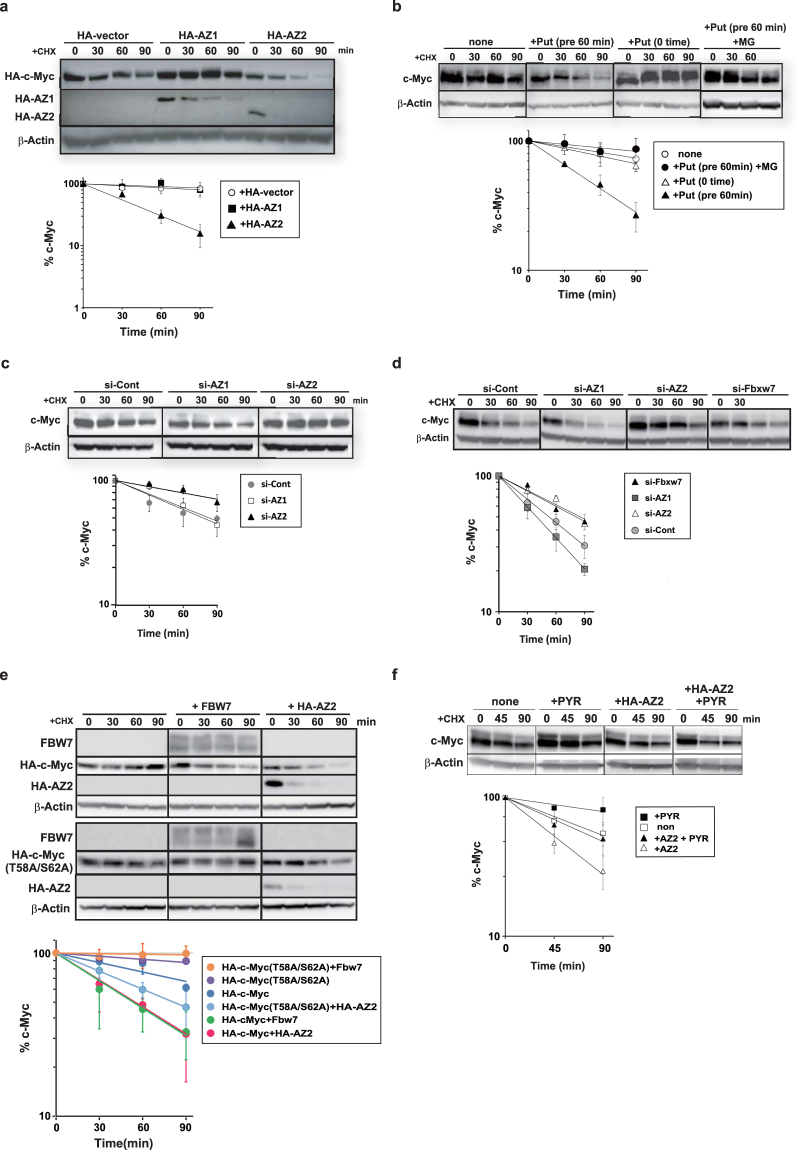
AZ2 accelerates c-Myc degradation by the proteasome without ubiquitination. (**a**) 293-F cells were cotransfected with HA-c-Myc and either HA vector, HA-AZ1, or HA-AZ2. After 24 h, cells were treated with 30 μg/ml cycloheximide and incubated for the indicated times. c-Myc and AZ levels in whole cell extracts were detected by immunoblotting using anti-HA antibody (upper panels). β-Actin bands are also shown. The percentage of c-Myc remaining was quantitated by image analysis and best-fit exponential lines are shown (bottom graph). (**b**) Putrescine (Put, final 2 mM) was added to the medium 60 min before or simultaneously with cycloheximide. Endogenous c-Myc level in whole cell extracts was detected by immunoblotting using antic-Myc antibody (upper panels). In one series, MG132 (20 µM) was also added 6 h before cycloheximide. (**c**) 293-F cells were transfected with control siRNA (50 nM) or AZ1 or AZ2 siRNA (20 nM). After 48 h, putrescine (2 mM) was added and after 60 min, c-Myc degradation assays were performed and shown as in (**b**). (**d**) Panc-1 cells were transfected with control siRNA (50 nM) or AZ1, AZ2, or Fbxw7 siRNA (20 nM). After 48 h, c-Myc degradation assays were performed and shown as in (**b**). (**e**) 293-F cells were transfected with plasmids with mouse cDNA of HA-c-Myc or HA-c-Myc (T58A/S62A) in combination either HA-AZ2 or HA-FBW7. Degradation assay of c-Myc and c-Myc (T58A/S62A) was performed and shown as in (**a**). (**f**) Untransfected 293-F cells or 293-F cells transfected with HA-AZ2 for 18 h were treated with E1 inhibitor, PYR-41 (40 μM) for 6 h. Endogenous c-Myc was detected as in (**b**) (upper panels). Data in a-f represent the mean ± SD calculated from three independent experiments.

A distinctive function of AZs is that its binding to ODC triggers ODC degradation by the proteasome without ubiquitination^10,14,15^. It has also been reported that AZ1 interacts with some proteins other than ODC to accelerate their degradation^23–25^. Therefore, we tested whether AZ2 accelerates c-Myc degradation in cells. AZ2 binds to and activates, ATP citrate lyase without affecting its stability^26^, but no protein other than ODC is known to be destabilized by AZ2. HA-tagged c-Myc (HA-c-Myc) was expressed in 293-F cells alone or together with HA-AZ2, and after addition of cyclohexamide, c-Myc levels were measured by immunoblotting using anti-HA antibody. As shown in Fig. 3a HA-AZ2 clearly accelerated degradation of HA-c-Myc whereas HA-AZ1 had no effect. We next examined the effect of the polyamine putrescine, an inducer of AZ2 (Fig. 3b). With pretreatment of cells with putrescine for 60 min before addition of cycloheximide, degradation of endogenous c-Myc was accelerated, and the acceleration was inhibited by MG132. This indicates that the degradation was catalyzed by the proteasome. It is noteworthy that when putrescine and cycloheximide were simultaneously added, c-Myc stability was unaffected, suggesting that the effect of putrescine is not direct but via inducing a protein factor, likely AZ2 (Fig. 3b) We performed AZ2-mediated degradation assays in the presence or absence of the lysosome inhibitors, leupeptin and chloroquine (data not shown) and the proteasome inhibitor, MG132 (Fig. S4). However, AZ2-mediated c-Myc degradation was inhibited only by MG132. To confirm the involvement of AZ2 in the putrescine-induced destabilization of c-Myc, we performed a knockdown of AZ2 using siRNA. Activities of the siRNAs were confirmed beforehand (Fig. S5a–c). In putrescine-treated 293-F cells, AZ2 siRNA significantly stabilized c-Myc compared to control siRNA (Fig. 3c). In contrast, AZ1 siRNA does not stabilize c-Myc. We observed that AZ1 siRNA increased putrescine and spermidine levels up to approximately 24- and 1.8-fold, respectively, and AZ2 siRNA slightly increased putrescine and spermidine levels up to approximately 2- and 1.5-fold, respectively in 293-F cells (Fig. S6). Essentially identical results for AZ2 siRNA were obtained in Panc-1 cells, although the destabilization of c-Myc by AZ1 siRNA is more evident (Fig. 3d). We suspect that in the cells treated with AZ1 siRNA, increased polyamines induced AZ2, which caused acceleration of c-Myc degradation. Next, we compared the effect of knockdown of AZ2 and E3 ubiquitin ligase Fbxw7. In both 293-F cells (result not shown) and Panc-1 cells (Fig. 3d) Fbxw7 siRNA resulted in suppression of c-Myc degradation to the same extent as AZ2 knockdown. To further confirm c-Myc destabilization activity of AZ2 *in vitro*, we performed a degradation assay of ^35^S-Methionine-labeled c-Myc (and ODC as a control) in rabbit reticulocyte lysates supplemented with an ATP regeneration system with or without, AZs (Fig. S7). As previously reported^14,16^, AZ1 accelerated ODC degradation in an energy-dependent and proteasome dependent manner. Meanwhile, AZ1 failed to accelerate c-Myc degradation *in vitro* confirming the result in cells (Fig. 3a). Under the same condition, AZ2 did not accelerate the degradation of ODC or c-Myc. Thus, the difference in AZ2’s ability to accelerate degradation of target proteins between *in vivo* and *in vitro* is a common feature for both ODC and c-Myc.

Sequential phosphorylation of Ser-62 and Thr-58 is a prerequisite for the ubiquitination of c-Myc with E3 ubiquitin ligase Fbxw7 recognizing the phosphorylated Thr-58 residue of c-Myc^5^. We examined degradation of HA-tagged c-Myc in which the two phosphorylation site residues were substituted with alanine (HA-c-Myc (T58A/S62A)) in 293-F cells (Fig. 3e). HA-tagged wild-type c-Myc was destabilized when coexpressed with either AZ2 or Fbw7. The mutant HA-c-Myc (T58A/S62A) was not destabilized by Fbw7 as previously reported^5^, but still destabilized by AZ2. Acceleration of degradation of HA-c-Myc (T58A/S62A) by AZ2 was also observed in the human osteosarcoma cell line U2OS (Fig. S8). To test whether AZ2-mediated c-Myc degradation is independent of ubiquitination, a cell-permeable inhibitor of E1 ubiquitin-activating enzyme, PYR41, was added to 293-F cells. PYR41 partially inhibited c-Myc degradation, but expression of AZ2 accelerated c-Myc degradation even in the presence of PYR41 (Fig. 3f). These results indicate that AZ2-madiated c-Myc degradation by the proteasome is ubiquitin independent.

### AZ2 contributes to pre-rRNA synthesis through the control of nucleolar c-Myc level

**Figure 4:**
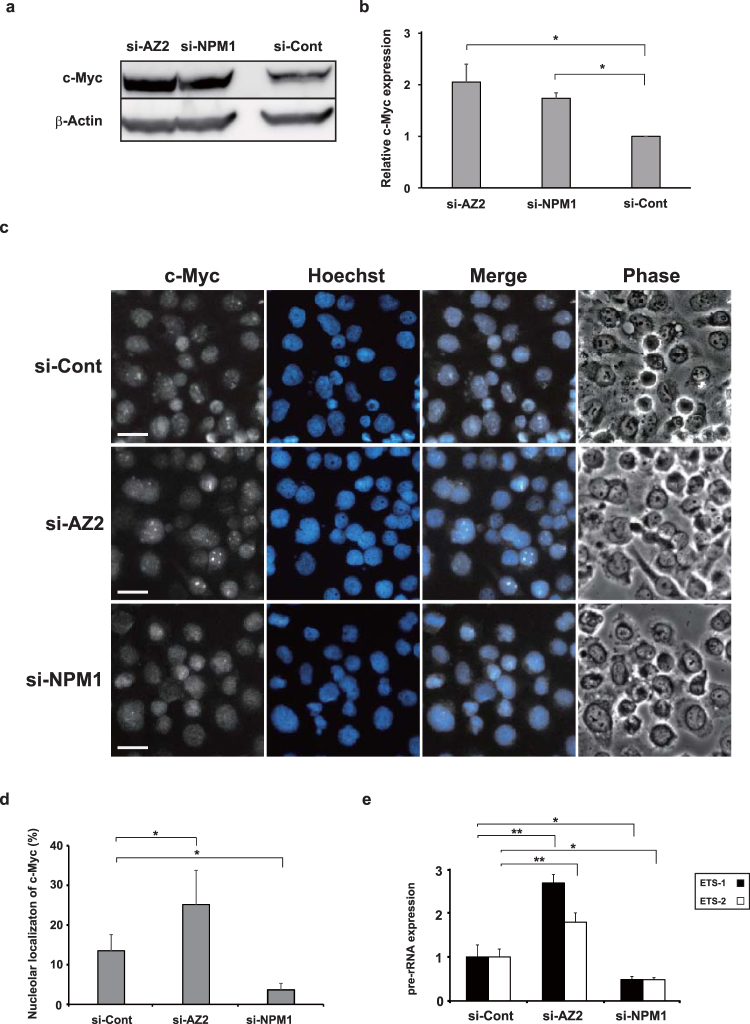
Knockdown of AZ2 increases nucleolar localization of c-Myc and stimulates pre rRNA expression. (**a**) Panc-1 cells were treated with either control, AZ2 or NPM1 siRNA and collected after 48 h. Endogenous c-Myc levels in the cell extracts were examined by immunoblotting using anti-c-Myc antibody. (**b**) Quantitative analysis of data in (**a**). c-Myc/β-actin ratio calculated from three independent experiments. (**c**) Panc-1 cells were treated with indicated siRNA for 48 h and then with MG132 for 60 min. Cells were fixed and endogenous c-Myc was visualized using anti-c-Myc antibody and secondary antibody conjugated with AlexaFluor 488. The brief treatment with MG132 was necessary to visualize nucleolar c-Myc. Distribution of c-Myc was displayed as monochrome images. Nuclei was stained with Hoechst 33342 and colored in cyan. Phase-contrast images were shown to confirm the location of nucleoli. Three independent experiments were performed. Scale bars, 20 μm. (**d**) Percentages of the cells in which nucleolar localization of c-Myc were observed. Three hundred cells were scored from random fields for each siRNA. (**e**) Panc-1 cells were treated with either control or AZ2 or NPM1 siRNA for 48 h and total RNA was prepared from the cells. qRT-PCR was performed using two probes of 47S pre-rRNA (ETS-1 and ETS-2) as described in Methods. Relative pre-rRNA expressions were represented with bar graph Data in b, d and e represent the mean ± SD calculated from three independent experiments. *P < 0.05, **P < 0.01 versus the control (t test).

Nucleolar c-Myc plays a key role in positively regulating ribosomal RNA (rRNA) synthesis^21^, with its requisite for nucleolar localization being promoted by a nucleolar protein nucleophosmin 1 (NPM1)^27^. Overexpression of NPM1 increases the nucleolar proportion of c-Myc and stimulates c-Myc-mediated rRNA synthesis whereas knockdown of NPM1 represses it. That NPM1 also destabilizes wild type and notably a T58A mutant of c-Myc is consistent with the finding that c-Myc is mainly degraded in the nucleolus^27^. Therefore, it is reasonable to hypothesize that AZ2 is involved in the NPM1-mediated regulation of c-Myc. To address this possibility, we examined the effect of knockdown of AZ2 or NPM1 on the nucleolar localization of c-Myc and rRNA biosynthesis. Treating Panc-1 cells with AZ2 or NPM1 siRNAs increased cellular c-Myc level two-fold (Fig. 4a,b). However, the cellular distribution of c-Myc after siRNA treatment displays a sharp contrast; the proportion of cells with nucleolar c-Myc doubled with AZ2 knockdown whereas it decreased to one-fourth with NPM1 knockdown (Fig. 4c,d). Expression of pre-rRNA that reflects rRNA synthesis, was analyzed by qRT-PCR with two probes complementary to the 5′ external transcribed spacer sequences of 47 S pre-rRNA (ETS-1 and ETS-2). The expression was also increased 2- to 3-fold in cells treated with AZ2 siRNA whereas it was decreased to a half with NPM1 siRNA (Fig. 4e).

### AZ2 is involved in the mechanism of c-Myc downregulation under the glucose-free or hypoxia conditions

**Figure 5:**
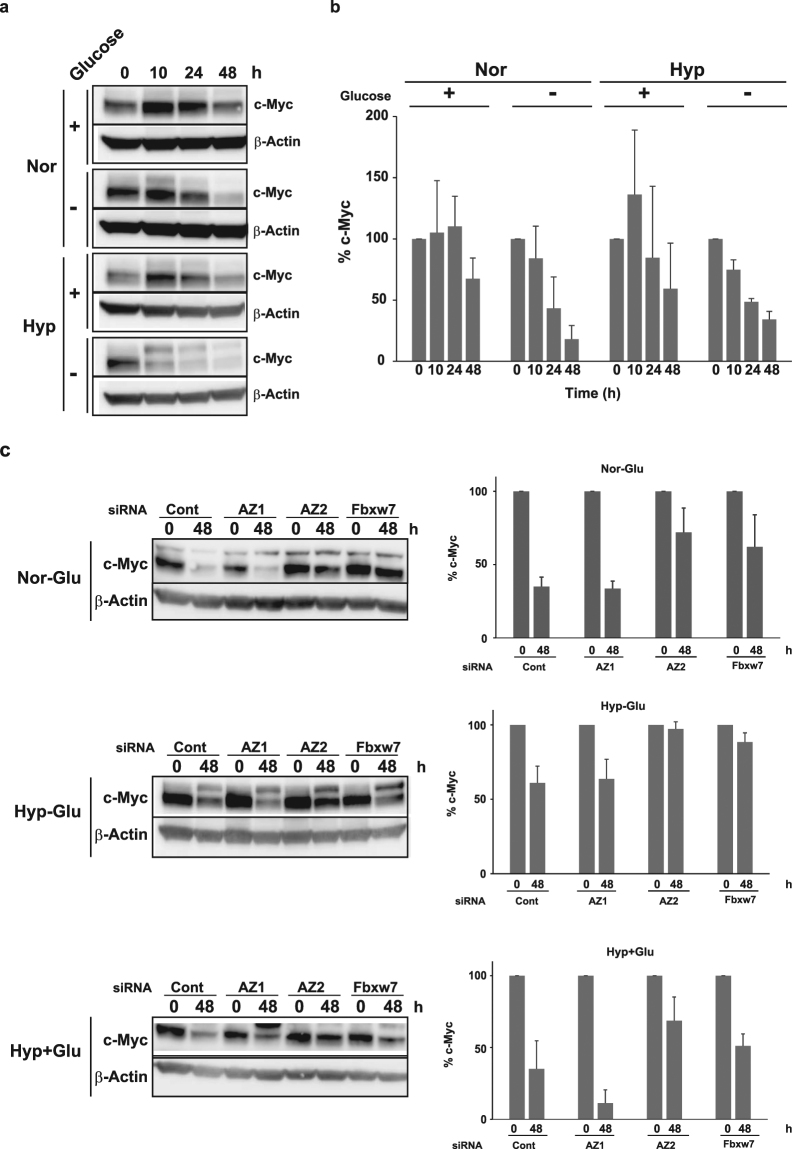
AZ2 contributes to downregulation of c-Myc under hypoxic and glucose free conditions. (**a**) Panc-1 ells were cultured under normal or hypoxic condition in medium with or without glucose. The cells were harvested at indicated times and endogenous c-Myc levels were analyzed by immunoblotting using anti c-Myc antibody. β-Actin bands are also shown. Nor, normoxia, Hyp, hypoxia. (**b**) Statistical analysis of (**a**). (**c**) Panc-1 cells were transfected with control, AZ1 or AZ2 siRNA (20 nM). After 24 h, the medium was changed to glucose-free medium and cells were cultured 48 h under hypoxic condition. Endogenous c-Myc levels were analyzed as in (**a**). Nor-Glu, normoxia and glucose-free, Hyp-Glu, hypoxia and glucose-free, Hyp + Glu, hypoxia in the presence of glucose, Data in a-c represent the mean ± SD calculated from three independent experiments.

**Figure 6:**
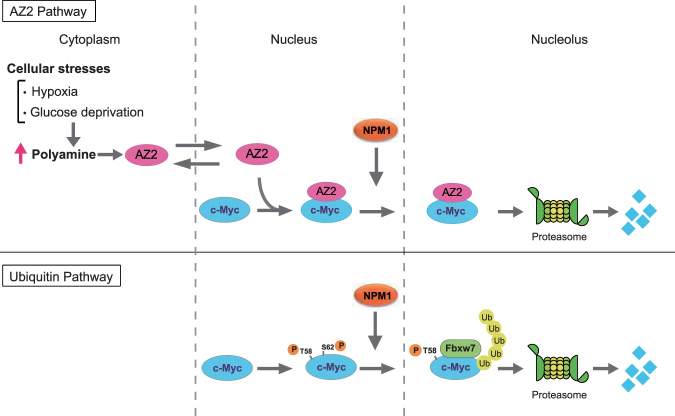
A model for AZ2-mediated c-Myc degradation in the nucleolus. Stress stimuli such as hypoxia and glucose deprivation increase cellular polyamine levels, resulting in induction of AZ2. AZ2 shuttles between cytoplasm and nucleus, and interacts with c-Myc in the nucleoplasm. c-Myc takes AZ2 to the nucleolus, where AZ2 accelerates c-Myc degradation by the proteasome without ubiquitination. This pathway is independent of the known pathway that requires Thr-58 phosphorylation and ubiquitination of c-Myc by Fbxw7 (Ubiquitin Pathway).

When does AZ2-dependent c-Myc degradation naturally occur? Okuyama *et al*. reported that hypoxia and glucose-free conditions downregulate c-Myc^28^. The mechanism of this downregulation is different from the previously reported HIF1α-induced mechanism that requires phosphorylation at Thr-58 following its ubiquitination by Fbxw7^29^. Since the hypoxic condition reportedly increases cellular polyamine levels^30^, we hypothesized that AZ2 might mediate c-Myc degradation under the glucose-free and/or hypoxic conditions. To test this hypothesis, Panc-1 cells were cultured under hypoxic (1% O_2_) and/or glucose-free conditions. Cellular hypoxic stress was confirmed by checking the induction of HIF1α protein (Fig. S9). When cells were cultured under the normoxic condition, the c-Myc level was slightly increased until 24 h and then decreased to 70% until 48 h, and under the glucose-free or both glucose-free and hypoxic condition, c-Myc was decreased to 40% and 50%, respectively until 24 h, and 20% and 35%, respectively until 48 h (Fig. 5a,b). Under the hypoxic condition with glucose-containing medium, c-Myc was increased to 150% until 10 h and then decreased to 60% until 48 h. The downregulation of c-Myc under these conditions except for normoxia was inhibited by the treatment of AZ2 or Fbxw7 siRNA for 48 h in Panc-1 cells (Fig. 5c). AZ1 siRNA does not inhibit c-Myc downregulation. Inversely further downregulation of c-Myc was observed only in the hypoxic condition and not in a control (Fig. 5c bottom bar graph). We measured polyamine concentration under these conditions to confirm the increase of cellular polyamine levels in Panc-1 cells (Fig. S10). Under the glucose-free condition, putrescine was increased five-fold until 10 h and spermidine was gradually increased 2.5-fold until 48 h but spermine was not changed. Under the hypoxic condition with glucose, putrescine was initially decreased, and then that increased two-fold until 48 h, and spermine was gradually increased 1.5-fold until 48 h, but inversely spermidine was decreased gradually. Under both glucose-free and hypoxic condition, only putrescine was increased 2.8-fold until 24 h, but spermidine was gradually decreased like hypoxic condition, and spermine was not changed. These results provide evidence that at least glucose-free or hypoxic conditions are environments in which AZ2 is induced and where it exerts its function by accelerating c-Myc degradation. Thus AZ2-madiated c-Myc downregulation is likely to be induced by stress condition like glucose-free or hypoxic condition (Fig. 6).

## Discussion

In the present study, we show for the first time that AZ2 interacts with c-Myc and accelerates c-Myc degradation by proteasomes (Figs 1 and 3). AZ2 colocalizes with c-Myc in the nucleus and nucleolus, and both proteins accumulate in the nucleoli in the presence of a proteasome inhibitor (Fig. 2). Prior work has shown that the E3 ubiquitin ligase Fbxw7 interacts with, and polyubiquitinates, c-Myc to promote its degradation^5^. Further, a non-ubiquitinated mutant of c-Myc can be degraded by the proteasome in the nucleolus^27^. These results suggest the existence of a local ubiquitin-independent degradation pathway for c-Myc^27^. Our results that AZ2 destabilizes the mutant c-Myc (T58A) and accelerates degradation of c-Myc in the presence of the E1 inhibitor (Fig. 3e,f) provide strong evidence that ubiquitin-independent c-Myc degradation is mediated by AZ2. Both pathways (Fig. 6) seem to contribute to regulating c-Myc degradation since knockdown of AZ2 and Fbxw7 by siRNA inhibit c-Myc degradation to the similar extent (Fig. 3d). However, we cannot exclude the possibility that additional factors may be required for AZ2-madiated c-Myc degradation because in our *in vitro* degradation assay, AZ2 purified from bacteria could not degrade c-Myc (Fig. S8). This may mean that the nucleolar localization of c-Myc and AZ2 is essential for AZ2-mediated c-Myc degradation. Actually, knockdown of AZ2 significantly increased the level of pre-rRNA by increasing nucleolus-localizing c-Myc (Fig. 4e). This suggests that AZ2 is likely involved in regulating ribosome biogenesis through c-Myc degradation. Further studies are needed to reveal the detailed mechanism.

We also demonstrated that downregulation of c-Myc under glucose-deprivation and/or hypoxic conditions, is partially mediated by AZ2 (Fig. 5c). It has been proposed that growth deceleration through c-Myc downregulation under such conditions may be a survival strategy of cancer cells^28^. In addition, under the glucose-free condition, rapid downregulation of c-Myc and rapid increase of putrescine and spermidine were observed (Fig. 5b,c and S10). This may also mean that AZ2-mediated c-Myc degradation is involved in malnutrition and starvation.

ODC, the key enzyme for polyamine biosynthesis, is a known transcriptional target of c-Myc^31^. Regulation of c-Myc by AZ2 and polyamines is a novel connection between the MYC signaling and polyamine metabolism, and might be a potential therapeutic target for controlling cancer. Since the molecular architecture of c-Myc is quite similar to MYCN^20^, which is critical for normal brain development, we speculate that AZ2 also controls degradation of MYCN. This could explain why AZ2-expression is correlated with the survival of neuroblastoma patients^19^.

## Methods

### Cell culture and transfection

Suspension 293-F cells (Life Technologies) were cultured in FreeStyle 293 Expression Medium (Life Technologies) or Expi293 Expression Medium (Life Technologies) in a 37 °C incubator under a humidified 5% CO_2_ atmosphere with Erlenmeyer flasks rotating at 135 rpm. Panc-1, HCT116, and U2OS cells were obtained from the European Collection of Authenticated Cell Cultures (ECACC). These cell lines were grown in Dulbecco’s modified Eagle’s medium (DMEM) supplemented with 10% fetal calf serum (FCS) and non-essential amino acid mixture (GIBCO) at 37 °C in a humidified atmosphere with 5% CO_2_. For the hypoxic condition, cells were grown in humidified nitrogen with 5% CO_2_ and 1% O_2_. The normal medium contains final 22.5 mM Glucose. Glucose-free DMEM was purchased from Sigma-Aldrich. The proteasome inhibitor MG132 (Peptide Institute) or E1 ubiquitin activating enzyme inhibitor PYR41 (Biogenova) was added to the medium at indicated times. For the glucose-free condition, glucose was omitted from the medium. For transfection of 293-F cells with plasmids, either 293fectin Transfection Reagent or ExpiFectamine 293 Transfection Kit (Life Technologies) was used. Transfection of Panc-1 and U2OS cells with plasmids was performed using Lipofectamine 2000 Transfection Reagent (Life Technologies). In the case of siRNA transfection, Lipofectamine™ RNAiMAX Transfection Reagent (Thermo Fisher Scientific) was used.

### Plasmid constructions

Human cDNAs for c-Myc, Fbxw7, ODC and NPM1 were amplified from the human brain cDNA library (Clontech) by polymerase chain reaction (PCR). Human AZ1ΔT and AZ2ΔT cDNAs were prepared as described for the mouse cDNAs^17^. These cDNAs were inserted into the *EcoR*I and *Kpn*I restriction sites of pCMV-HA vector (Clontech) to generate pCMV-HA-h-c-Myc, pCMV-HA-Fbxw7, pCMV-HA-h-ODC, pCMV-HA-h-NPM1, pCMV-HA-h-AZ1, pCMV-HA-h-AZ2, respectively. Mouse c-Myc and FBW7 (Fbxw7) cDNA were amplified from the mouse kidney cDNA library (Clontech) by PCR and were inserted into the *EcoR*I and *BamH*I restriction sites of pCMV-HA vector to generate pCMV-HA-c-Myc and pCMV-HA-FBW7, respectively. Mouse c-Myc cDNA was inserted into the *EcoR*I/*BamH*I restriction sites of pEYFP vector to generate pEYFP-c-Myc. Subcellular localization were analyzed using plasmid pEYFP-c-Myc, pECFP-AZ2^17^, pCMV-HA-h-AZ2 and pCMV-HA-h-ODC. Plasmid p3xFLAG-ODC^17^ was used for expression of FLAG-tagged mouse ODC. To generate plasmids p3xFLAG-AZ1, p3xFLAG-AZ2, and p3xFLAG-c-Myc, DNA fragments encoding mouse AZ1ΔT, AZ2ΔT, and c-Myc were amplified by PCR and inserted into *Eco*RI/*Xba*I sites of the p3xFLAG-CMV-7.1 vector (Sigma). cDNAs for the phosphorylation site mutants of mouse (T58A/S62A) and human (T58A) were amplified by PCR with primers corresponding to the mutant sequences and inserted into *Eco*RI/*BamH*I sites of pCMV-HA vector to generate pCMV-HA-c-Myc (T58A/S62A) and pCMV-HA-h-c-Myc (T58A), respectively. All constructs were verified by sequencing with an ABI PRISM 3700 sequencer and Big Dye terminator v3.1 cycle-sequencing kit (ABI). All plasmids for transfection were purified by Plasmid Midi Kit (Qiagen).

### Antibodies and immunoblot analyses

Anti-HA polyclonal and monoclonal antibodies were purchased from Medical & Biological Laboratories (MBL) and Cell Signaling Technology, respectively. Anti-FLAG antibody (M2) (Sigma). Anti-c-Myc rabbit monoclonal antibodies were purchased from Epitomics (c-Myc N-term) and abcam (Y69, ab32072) Anti-fibrillarin monoclonal antibody and anti-β-Actin polyclonal antibody were purchased from Cell Signaling Technology. Cells were washed twice in phosphate-buffered saline (PBS) and suspended in CelLytic M Cell Lysis Reagent (Sigma) supplemented with Protease Inhibitor Cocktail Set II (Calbiochem). The cell suspension was sonicated with a small size homogenizer Sonifier 250D (Branson) for 15 s (output 6) and centrifuged at 13,000 × *g* at 4 °C for 20 min. Protein concentrations of the supernatants were determined using Pierce BCA Protein Assay Kit (Thermo Fisher Scientific). The supernatants containing 50–70 μg of protein were separated by SDS-PAGE and protein bands were electrotransferred to Hybond-P (GE Healthcare) or Immobilon-P PVDF membrane (Millipore). Immunodetection was carried out with anti-HA and anti-FLAG polyclonal antibodies at dilutions of 1:2,000 and 1:40,000, respectively. Anti-rabbit IgG-conjugated horseradish peroxidase (GE Healthcare) was used as the secondary antibody at dilutions of 1:20,000. The proteins were visualized using ImmunoStar LD (Wako) and detected by LAS 400mini (GE Healthcare). Adobe Photoshop Elements 13 and Illustrator CC 2015 were used for processing images.

### Immunoprecipitation analysis

293-F cells expressing both HA-tagged and FLAG-tagged proteins were washed twice in phosphate-buffered saline (PBS). Cells were lysed in M-PER (Mammalian Protein Extraction Reagent, Thermo Fisher Scientific) supplemented with protease inhibitor cocktail Set II at room temperature for 15 min and centrifuged at 13,000 × *g* at 4 °C for 25 min. Supernatants were subjected to an immunoprecipitation analysis..Immunoprecipitaion was performed with anti-FLAG antibody (M2)-agarose at 4 °C overnight, and c-Myc, bound proteins were detected with anti-HA antibody by immunoblotting. Expressed protein levels in cell lysates (Input) were checked by immunoblotting with anti-HA or anti-FLAG antibody. Experiments were repeated three times.

### *In vitro* immunoprecipitation assay

Human HA-AZ2, HA-c-Myc or HA-ODC was expressed in 293-F cells, and these proteins were purified with HA-tag immunoaffinity column (HA-tagged Protein Purification Kit, MBL) following the manufacturer’s protocol. After exchanging the buffer to M-PER (Mammalian Protein Extraction Reagent, Thermo Fisher Scientific) by centrifuge using ultrafiltration membrane, Ultrafree-0.5 (molecular weight limit 10 kD, MILLIPORE), the purified proteins (approximately 100 ng each) were mixed in M-PER, and then anti c-Myc antibody and Dynabeads Protein G magnetic beads (Life Technologies) were added and incubated at 4 °C overnight. After washing the mixture 5 times with M-PER by magnetic rack. c-Myc bound proteins were eluted with SDS-PAGE sample buffer and subjected to SDS-PAGE (12.5%.gel). c-Myc bound proteins were detected with anti-HA antibody by immunoblotting. Experiments were repeated twice.

### Fluorescence microscopy

Panc-1 cells expressing ECFP and EYFP fusion proteins were visualized by an IX70 inverted fluorescence microscope (Olympus) equipped with an ECFP and EYFP specific fluorescence mirror unit (Olympus), CoolSNAP EZ CCD Camera (Photometorics) and MetaVue imaging software (Molecular Devices). Nuclear DNA was stained with 100 ng/ml of Hoechst 33342 (Molecular Probes) for 90 min under the culture conditions. After staining, cells were washed twice with fresh medium, and incubated for 1 h before observation. A fluorescence mirror unit U-MNU2 (Olympus) was used to visualize nuclear stained with Hoechst 33342. Nucleoli were immunostained with anti-fibrillarin antibody and monoclonal anti-rabbit IgG conjugated with AlexaFluoro-555 (Molecular Probes). For immunocytochemical detection of HA-tagged proteins, cells were seeded on glass-bottom plates and transfected as above. Twenty-four hours after transfection, cells were fixed with methanol and acetone pre-chilled at −20 °C for 10 min and 1 min, respectively. Fixed cells were incubated with anti-HA monoclonal antibody or diluted 1:500 in blocking solution (PBS, 5% BSA) at room temperature for 1 h or at 4 °C overnight. HA-tagged proteins were detected using polyclonal anti-HA antibody and monoclonal anti-rabbit IgG antibody conjugated with Alexafluoro-555 fluorescence probes (Molecular Probes). Alexafluoro-555 bound HA-tagged proteins were visualized by IX70 inverted fluorescence microscope with fluorescence mirror unit U-MWIG (Olympus). Adobe Photoshop Elements 13 and Illustrator CC 2015 were used for processing images.

### RNA interference

Cells were transiently transfected with 20–50 nM (AZ1, AZ2, and Fbxw7) or 100 nM (c-Myc and NPM1) siRNA. The following siRNAs were used: Human AZ1, MISSION SASI_Hs01_00086270 (Sigma); Human AZ2, MISSION SASI_Hs01_00196643 (Sigma); Human Fbxw7, siGENOME SMART pool M-004264–02–0005 (Dharmacon); and c-Myc, SignalSilence #6341 (Cell Signaling Technology); and Human NPM1, MISSION EHU115611-20UG (Sigma). All siRNAs are designed to minimize the off-target effects. Randomized negative control siRNA (Cosmo Bio) was used in all the RNA interference experiments. AZ2 siRNA used in this study was selected from three siRNAs (MISSION SASI_Hs01_00196641, SASI_Hs01_00196642, SASI_Hs01_00196643, Sigma). Mutation in AZ2 siRNA sequence (SASI_Hs01_00196643) suppressed the effect of c-Myc stabilization (Fig. S3c). Lipofectamine RNAiMAX transfection reagent (Thermo Fisher Scientific) was used for transfection of siRNA according to the manufacturer’s instructions.

### qRT-PCR

Total RNA was purified from 293-F or Panc-1 cells using TRIzol LS Reagent (Thermo Fisher Scientific) and Direct-zol RNA MiniPrep (ZYMO RESEARCH). cDNA was synthesized from 2 μg of purified total RNA using Superscript VILO cDNA synthesis kit (Thermo Fisher Scientific) according to the manufacturer’s protocol. qRT-PCR was performed with single tube TaqMan Gene Expression Assays using a StepOnePlus real-time PCR system (Thermo Fisher Scientific). The following probe and primer sets were used: AZ1 (Hs00427923_m1), AZ2 (00159726_m1), c-Myc (Hs00153408_ml), Fbxw7 (Hs00217794_ml), and GAPDH Control (402869) or Eukaryotic 18 S rRNA Endogenous Control (4333760 F). For pre-rRNA expression analysis, two sets of Taq-Man probes and primers to detect and amplify 5′ external transcribed spacer (ETS-1 and ETS-2) of human 47 S pre-rRNA were designed by Custom TaqMan Assay Design Tool (Thermo Fisher Scientific) as follows: ETS-1, Probe 5′-FAM-TAGCCGGCCGCGCT-3′, Primers 5′-TCTGGCCTACCGGTGACC-3′ and 5′-GCAGGCGGCTCAAGCA-3′, For ETS-2, Probe 5′-FAM-TCTAGCGATCTGAGAGGCGT-3′, Primers 5′-CCTTCCCCAGGCGTCCCTC-3′ and 5′-GGCAGCGCTACCATAACGGA-3′.

### c-Myc degradation assay *in vivo*

Suspension 293-F cells were transfected with pCMV-HA-c-Myc or pCMV-HA-c-Myc (T58A/S62A) either alone or in combination with pCMV-HA-AZ1, pCMV-HA-AZ2 or pCMV-HA-FBW7. For endogenous c-Myc degradation assay, the cells were transfected with pCMV-h-HA-AZ1 or pCMV-h-HA-AZ2. Twenty four hours after transfection, cycloheximide (30–50 μg/ml) was added to the medium and aliquot of cells were harvested at indicated time points by centrifugation at 1,800 rpm for 3 min. The cells were lysed with CelLytic M Cell Lysis Reagent (Sigma) or RIPA buffer (20 mM Tris-HCl, pH 7.6, 150 mM NaCl, 2 mM EDTA, 1% NP-40, 0.1% sodium deoxycholate, 0.1% SDS, and 50 mM sodium fluoride). HA-tagged c-Myc and endogenous c-Myc were detected by immunoblot analysis using anti-HA polyclonal antibody and anti-c-Myc antibody, respectively. In the case of adherent Panc-1 or U2OS cells, the cells t were cultured in ϕ35 mm plastic dish up to 80% confluence and were transfected with the plasmids mentioned above.

### Measurement of cellular polyamines

Cells were washed twice with ice-cold PBS and suspended in RIPA buffer supplemented with protease inhibitor cocktail set II (Merck Millipore). Cell lysates were placed on ice for 15 min, sonicated for 12 s, and centrifuged at 15,000 rpm for 25 min at 4 °C. Aliquots of supernatants were used to determine protein concentrations, and the rest were used for the measurement of polyamines. Trichloroacetic acid was added at a final concentration of 4% to the cell lysates, vortexed, and centrifuged for 20 min at 4 °C. The supernatants were filtrated by Millex-LH 0.45 μm (Merck Millipore) and subjected to HPLC analysis using Shimadzu LC Solution System (LC-20AT, SIL-20AC, CTO-20A, and RF-10AXL) equipped with a cation exchange column Shim-Pack ISC-05 (Shimadzu)^32^. Polyamine concentrations were calculated from each polyamine peak area compared with the standards (putrescine, spermidine and spermine).

## Electronic supplementary material

Supplemental Figure 1–10
