# Supplementary material for: Novel ubiquitin-independent nucleolar c-Myc degradation pathway mediated by antizyme 2
Source: Sci Rep. 2018 Feb 14;8:3005. doi: 10.1038/s41598-018-21189-0 (PMC5813005; doi:10.1038/s41598-018-21189-0)
Supplement: Supplementary file 1 — Supplemental Figure 1–10 [file 41598_2018_21189_MOESM1_ESM.pdf]

Novel ubiquitin-independent nucleolar c-Myc degradation pathway mediated by antizyme 2

Supplementary Figures

Noriyuki Murai, Yasuko Murakami, Ayasa Tajima & Senya Matsufuji

Department of Molecular Biology, The Jikei University School of Medicine

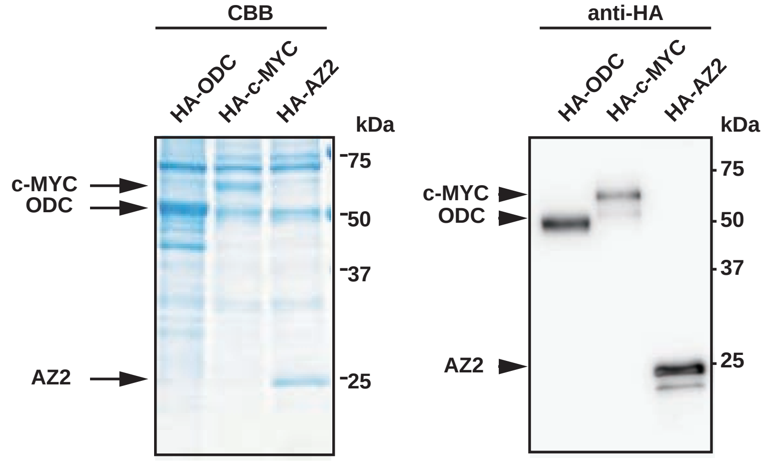

Supplementaly Figure S1. Purification of HA tagged c-Myc, AZ2 and ODC.
HA-tagged c-Myc, AZ2 and ODC (HA-c-Myc, HA-AZ2, HA-ODC) were expressed in 293-F cells. These proteins were purified using HA tagged Protein Purification Kits (MBL), from separate cell lysates in which each protein was expressed. 1/10 volume of the purified protein samples were analyzed by SDS-PAGE (12.5 % gel, left). The gel was stained with Coomassie Brilliant Blue (CBB). Immunoblot analysis of purified HA-tagged proteins by anti-HA antibody (anti-HA). Proteins of interest are indicated as arrows.

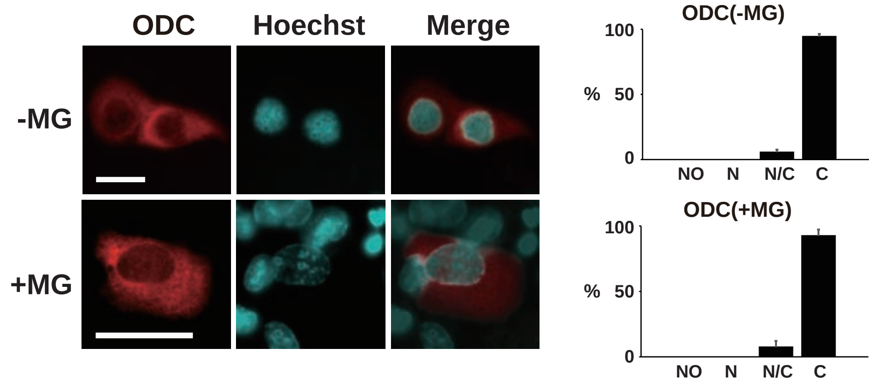

Supplementaly Figure S2. Subcellular localization of ODC in the presence and absence of MG132.
Panc-1 cells were transfected with HA-ODC (human) and then treated with (+MG) or without (-MG), MG132 for 5 h. ODC was immunostained with anti-HA antibody and AlexaFluor555-conjugated anti-rabbit IgG. Monochrome images of ODC and nuclei (Hoechst 33342) are colored in orange and cyan, respectively. Bar graphs on the right represent quantification of the cells with subcellular localization of ODC. Bar graphs on the right of each image represent quantification of 100 cells. Data shown represent the mean ± SD calculated from three independent experiments. Scale bars, 20 μm. NO, nucleolar dominant distribution, N, nuclear dominant distribution; N/C, nuclear and cytosolic distribution, C, cytosolic dominant distribution.

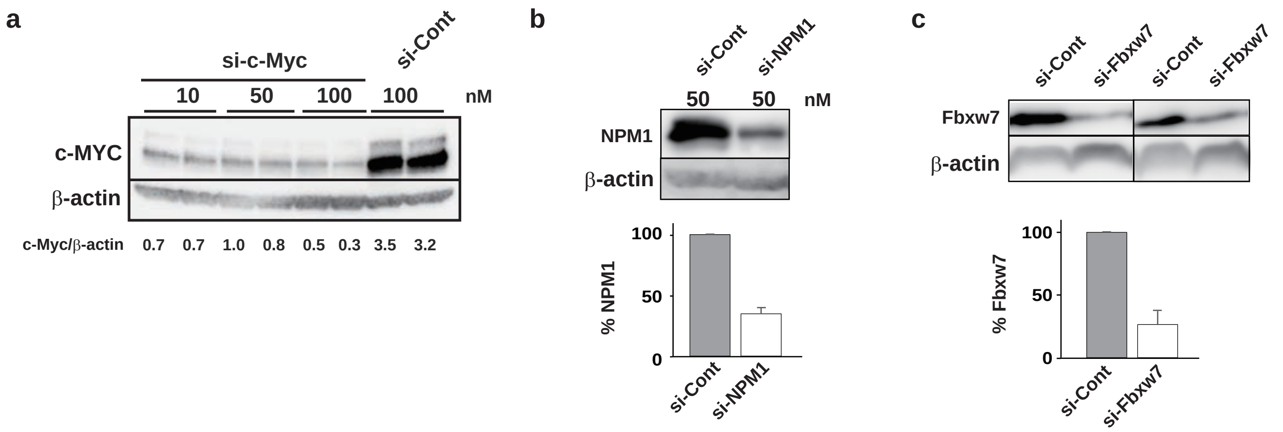

Supplementaly Figure S3. Knockdown of c-Myc, NPM1 and Fbxw7. (a) Panc-1 cells were treated with c-Myc siRNA or control siRNA at the indicated concentrations for 48 h. Endogenous c-Myc in the cell extract was detected with immunoblotting using anti-c-Myc antibody. c-Myc/β–actin ratios are indicated at the bottom of each band. (b) Panc-1 cells were treated with 50 nM of NPM1 siRNA or control siRNA at the indicated concentrations for 48 h. NPM1 was detected with immunoblotting using anit-NPM1 antibody. (c) Panc-1 cells were treated with 20 nM of Fbxw7 siRNA or control siRNA at the indicated concentrations for 48 h. Fbxw7 was detected by immunoblotting using anit-Fbxw7 antibody. Bar graphs in b and c represent the mean ± SD calculated from three independent experiments.

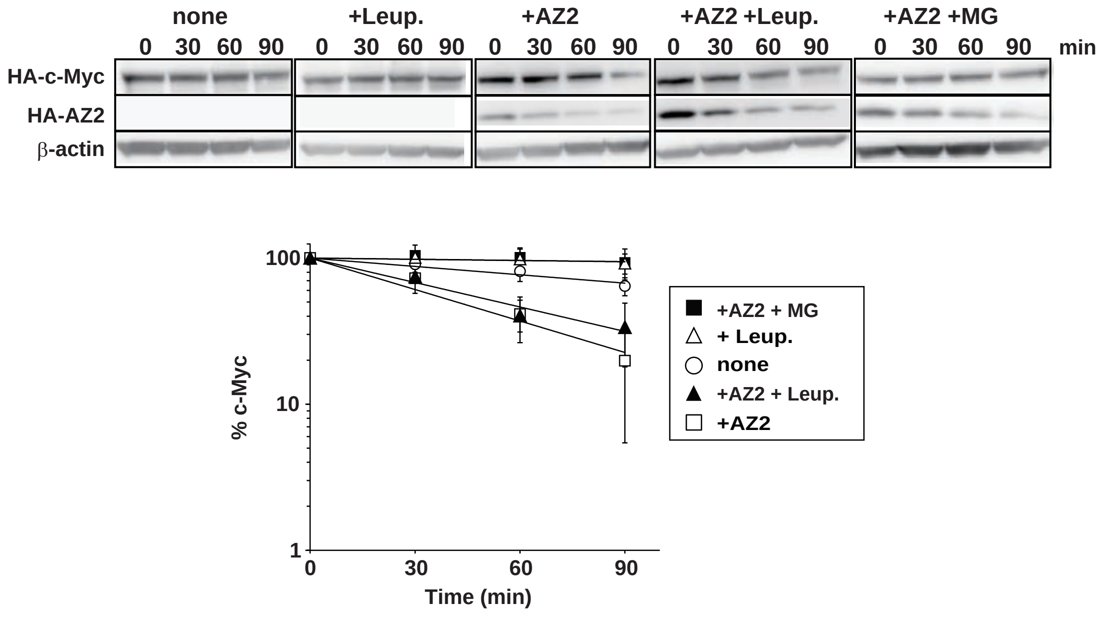

Supplementaly Figure S4. Effect of lysosome and proteasome inhibitor on AZ2-mediated c-Myc degradation. HA-c-Myc was expressed with or without either HA-vector control or HA-AZ2 in Panc-1 cells. 24 h after transfection, cells were treated with 50 μg/ml of leupeptin (+Leup.) or 10 μM of MG132 (+MG) for 6 h. The degradation assay was started by adding 50 μg/ml of cycloheximide and cells were collected at the indicated time. HA-c-Myc and HA-AZ2 were detected by immunoblotting using anti-HA antibody. Bar graph represent the mean ± SD calculated from three independent experiments.

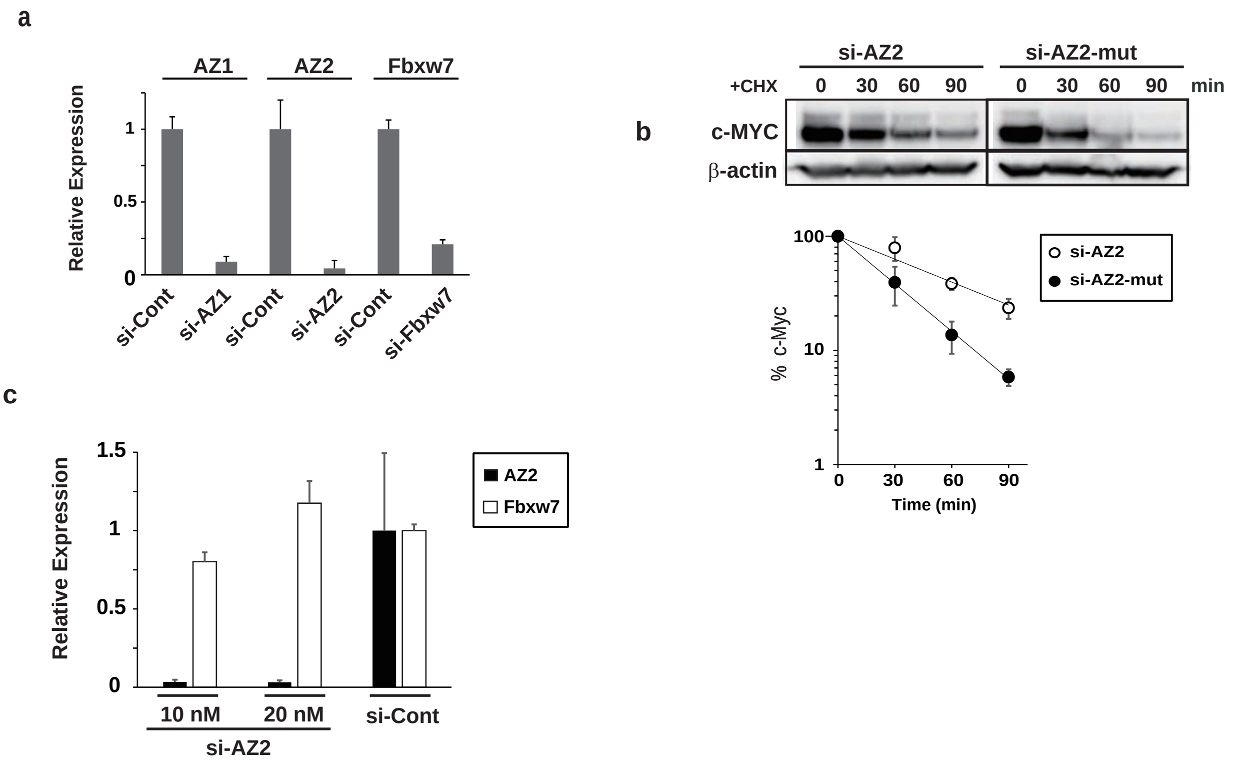

Supplementaly Figure 5. Knockdown of AZ2 in Panc-1 cells. (a) Panc-1 cells were transfected with siRNAs for AZ1, AZ2, Fbxw7 (20 nM) or control siRNA (50 nM) for 48 h, then AZ1, AZ2 and Fbxw7 mRNAs were measured by qRT-PCR. (b) Cells were treated with 20 nM of si-AZ2 (sense-strand sequence: 5' -GAUGUGAUGUUCAUGGUUUTT-3') or si-AZ2-mut (sense-strand sequence: 5' -GACGUAAUGUUAAUGGUCUTT-3') (mutated nucleotides are underlined) for 48 h. c-Myc was detected with immunoblotting and the percentage of c-Myc remaining was quantitated by image analysis. Best-fit exponential lines are shown (bottom graph). (c) Cells were treated with AZ2 siRNA (10 or 20 nM) or control siRNA (50 nM) for 48 h. Expression of AZ2 and Fbxw7 mRNA were measured by qRT-PCR. Data in a, b (bottom) and c represent the mean ± SD calculated from three independent experiments.

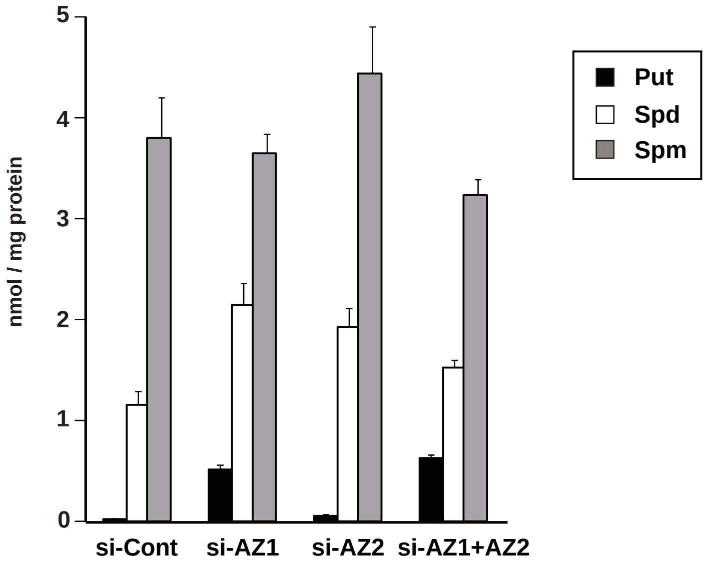

Supplementaly Figure 6. Polyamine content in 293-F cells treated with AZ siRNAs. 293-F cells were treated with AZ1 or AZ2 siRNA (20 nM) or both (20 nM each) or control siRNA (20 nM) for 48 h. Cellular polyamine concentrations were measured as described in Methods. Data shown represent the mean ± SD calculated from three independent experiments.

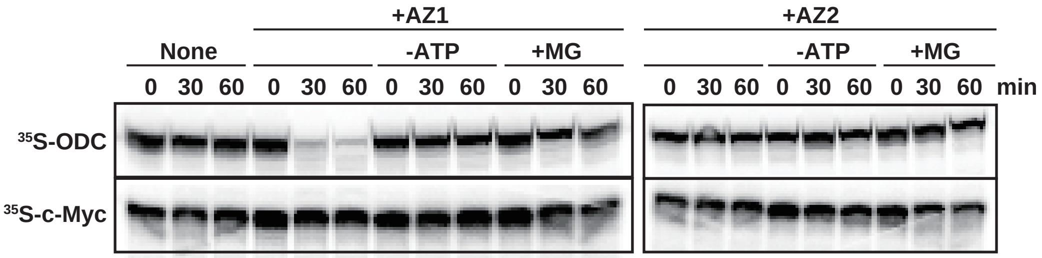

Supplementaly Figure 7. Effects of AZ1 and AZ2 on the degradation of ODC and c-Myc in the reticulocytes lysate. [35S]Methionine-labeled ODC, c-Myc, AZ1 and AZ2 were synthesized in TNT Quick Coupled Transcription/Translation system (Promega). Unpurified, labeled ODC or c-Myc were mixed with either AZ1 or AZ2 at a molar ratio of approximately 1: 2 in 35 μl of buffer containing ATP regeneration system (30 mM Tris-HCl, pH 7.5, 2 mM ATP, 10 mM $MgCl_2$, 2.5 mM dithiothreitol, 0.4 mM phosphocreatine, 0.2 mg/ml creatine kinase), then 15 μl of rabbit reticulocyte lysate (Promega) was added and incubated at 37℃. At the indicated times, 10 μl of mixture was collected and analyzed by SDS-PAGE followed by autoradiography. In –ATP condition, ATP was omitted from and EDTA (10 mM) was added to the mixture. In +MG condition, MG132 was added to the mixture at 50 μg/ml. The experiments were repeated twice.

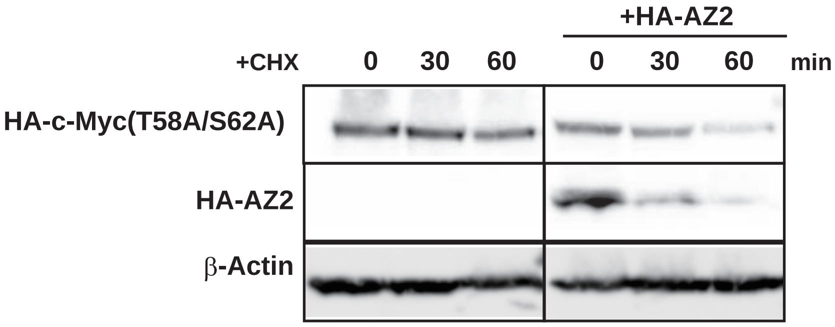

Supplementaly Figure 8. Degradation of ubiquitination mutant of c-Myc mediated by AZ2.

HA-c-Myc or HA-c-Myc (T58A/S62A) was expressed in U2OS cells with or without HA-AZ2. Degradation assay was performed as in Figure 3e. The experiment was repeated twice.

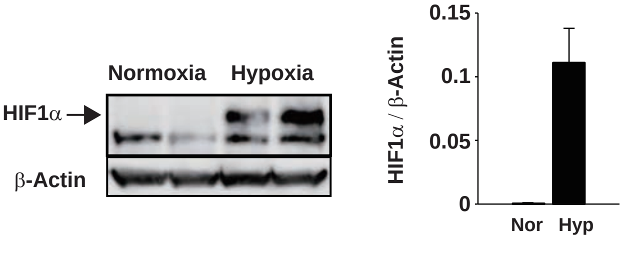

Supplementaly Figure 9. HIF1α expression in Panc-1 cells under the hypoxic condition. Panc-1 cells were cultured at normal or hypoxic (1% O_2) conditions for 48 h, and cell extracts were detected with immunoblotting using anti-HIF1α antibody. Bar graph of HIF1α/β-Actin ratios represents the mean ± SD calculated from three independent experiments.

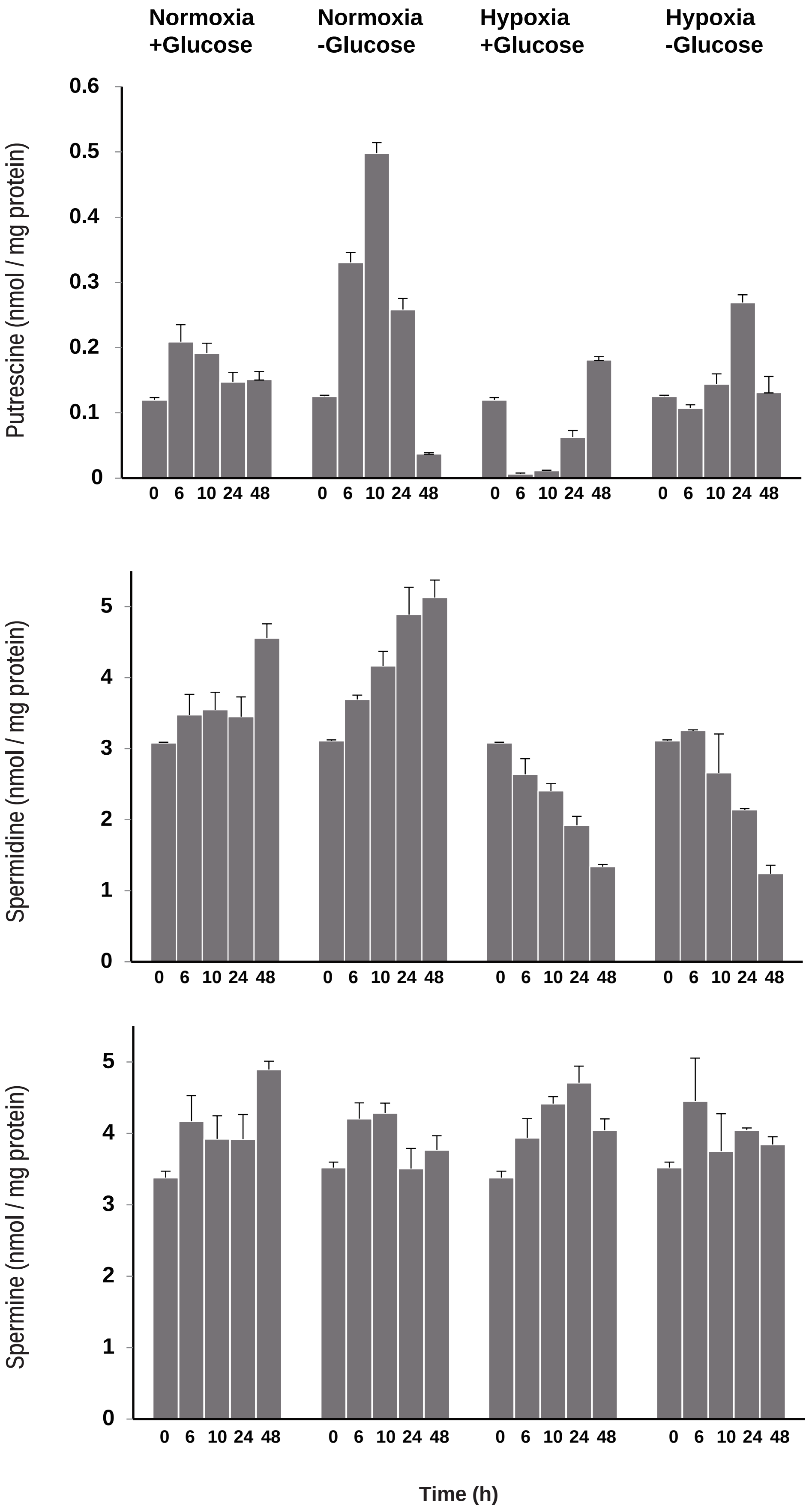

Supplementaly Figure 10. Polyamine contents of Panc-1 cells under various culture conditions.
The cells were cultured under the normal or hypoxic (1% O_2) condition and in medium with or without 22.5 mM glucose. Cellular polyamine concentrations were measured as described in Methods. The polyamine concentration data shown represent the mean ± SD calculated from three independent experiments.
